# Supplementary material for: Upregulation of large myelin protein zero leads to Charcot–Marie–Tooth disease-like neuropathy in mice
Source: Commun Biol. 2020 Mar 13;3:121. doi: 10.1038/s42003-020-0854-z (PMC7070019; doi:10.1038/s42003-020-0854-z)
Supplement: Supplementary file 1 — Supplementary Information [file 42003_2020_854_MOESM1_ESM.pdf]

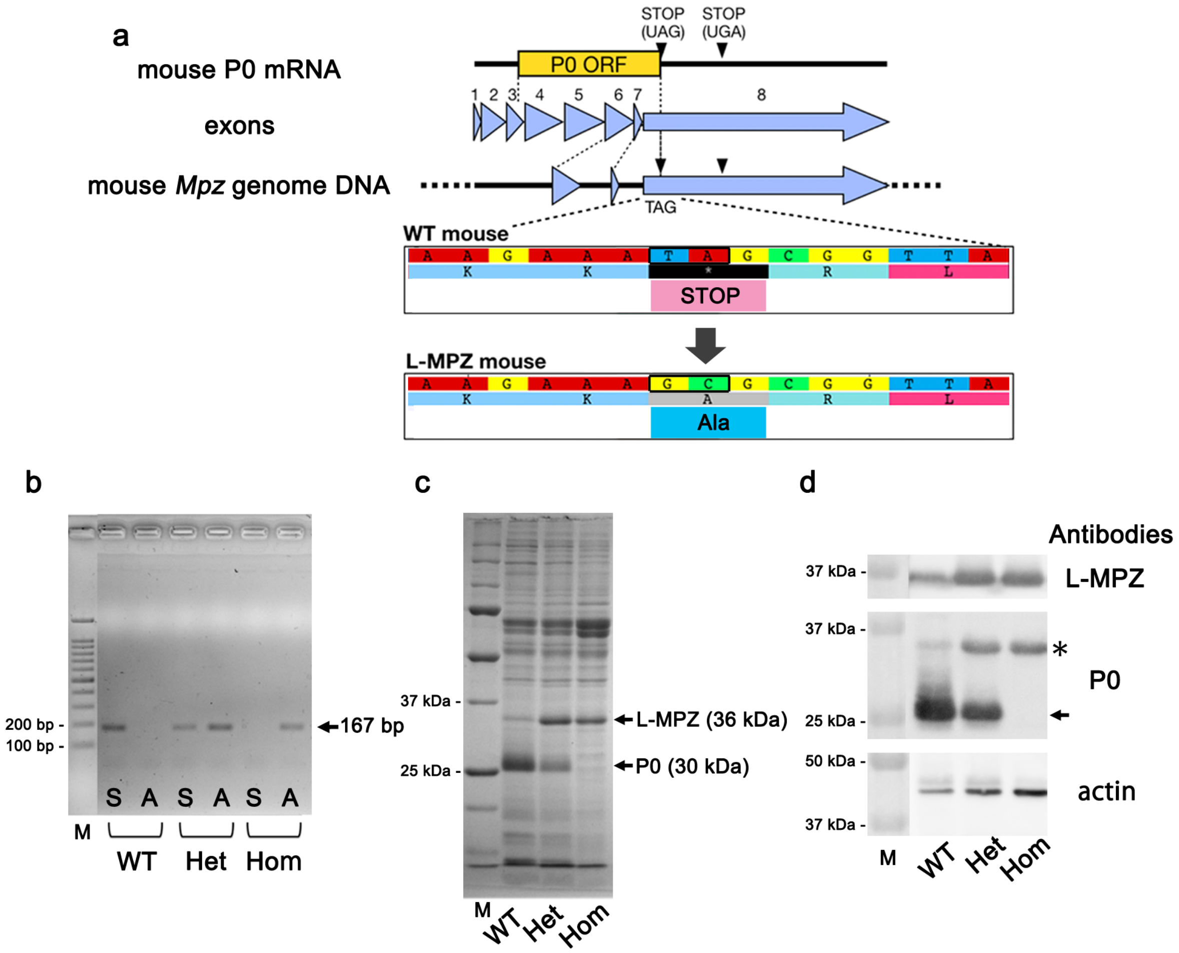

Supplementary Figure 1. Generation of L-MPZ mice by CRISPR–Cas9 system

(a) Sequences of the *Mpz* gene around the canonical stop codon and its substituted alanine codon in wild-type (WT) and L-MPZ mice, respectively. Replacement of TGA (stop codon) with GCG (alanine codon) was performed using the CRISPR–Cas9 system. (b) Allele-specific PCR of tail genomic DNA for genotyping of each mouse. The S primer set (S) (described in the Methods) generated a 167 bp PCR product from WT and heterozygous (Het) genomic DNA but not from homozygous (Hom) DNA. The A primer set (A) generated a 167 bp PCR product from Het and Hom genomic DNA but not from WT DNA. Allele-specific PCR using two primer sets successfully distinguished individual genotypes. M, marker. (c) Representative 12% SDS–PAGE separating three genotype nerve samples (5 μg per lane) stained by Coomassie Brilliant Blue. Amount of L-MPZ was approximately 10% of P0 in WT mice. As expected, the P0 band completely disappeared and L-MPZ levels increased in L-MPZ Hom mice, while decreased P0 and increased L-MPZ were observed in L-MPZ Het mice. M, marker. (d) Western blotting of sciatic nerve homogenates (5 μg per lane) in 10-week-old L-MPZ mice using antibodies against L-MPZ, P0 and actin. Note that the anti-P0 antibody used in this study recognizes both L-MPZ (asterisk) and P0 (arrow). M, marker. Uncropped blots for (d) can be found in Supplementary Fig. 10a–c.

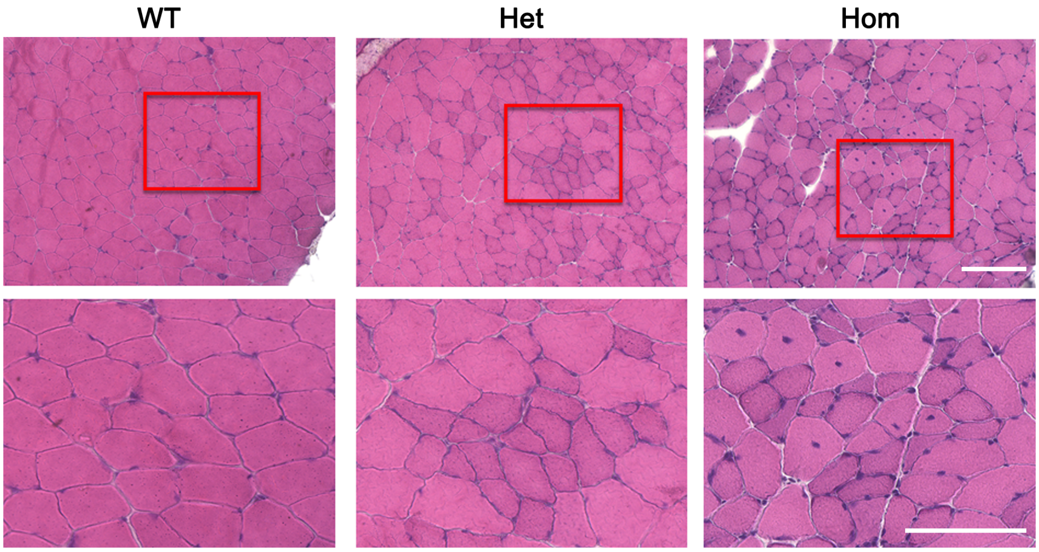

Supplementary Figure 2. Neurogenic muscular atrophy in L-MPZ mice
Hematoxylin and eosin staining pictures of peroneus muscle transverse sections from 4-month-old L-MPZ WT, Het, and Hom mice. Indicated red square areas were magnified in the lower row. In L-MPZ Hom and Het mice, grouped muscle fiber atrophies were observed. In addition, multiple muscle fibers displayed the central nuclei in L-MPZ Hom mice. Bar, 100 μm.

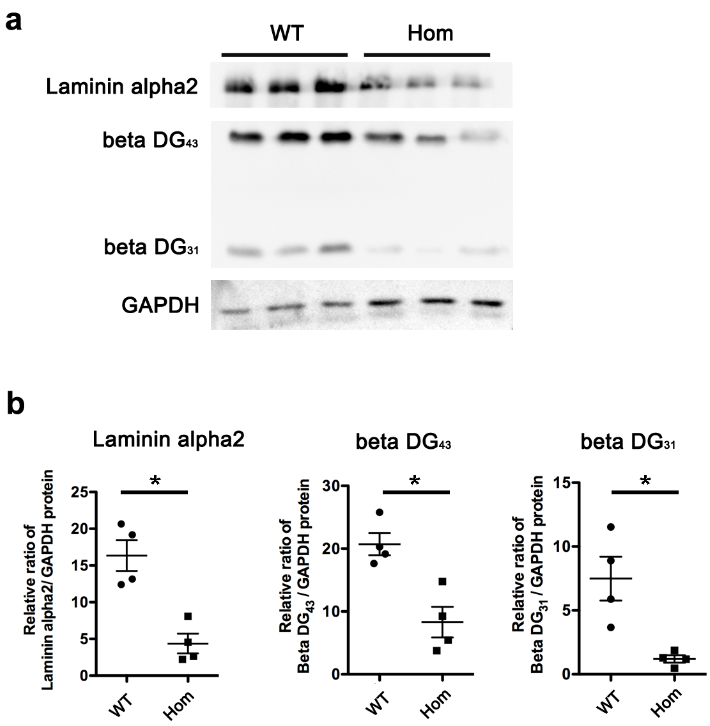

Supplementary Figure 3. Decrease of extracellular matrix-related proteins in L-MPZ mice

(a) Western blotting of sciatic nerve homogenates (5 μg per lane) in 10-week-old L-MPZ Hom mice using antibodies against anti-laminin alpha-2 and two subtypes of beta dystroglycan (DG_{31}, DG_{43}). (b) Quantification of band intensities from each of four individual mice. *p<0.05 by one-way ANOVA with post-hoc Tukey's test. Data are presented as mean ± SE of experiments. Uncropped blots for (a) can be found in Supplementary Fig. 10d–f.

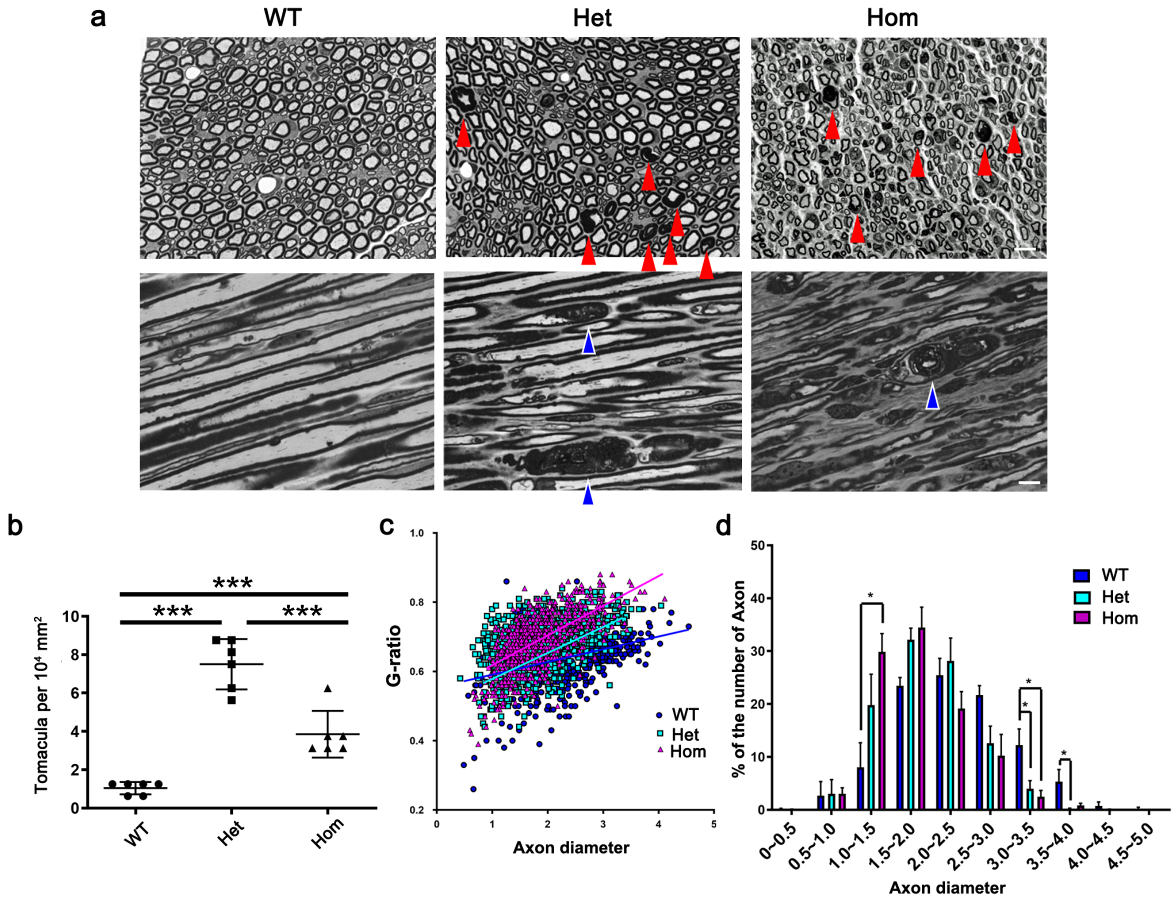

Supplementary Figure 4. Morphological analysis of semi-thin sections in L-MPZ sciatic nerves

(a) Toluidine blue staining of 500 nm-thick transverse and sagittal sections of sciatic nerves in 8-week-old L-MPZ WT, Het and Hom mice. Tomacula-like abnormalities in myelin were observed both in transverse and sagittal sections of Het and Hom mice (red and blue arrowheads). Bar, 10 μm. (b) For quantification, the number of tomacula per 10^4 μm^2 was counted in each sciatic nerve section of L-MPZ mice. These numbers were increased in L-MPZ Het and Hom mice. (c) The G-ratios (the ratio of axonal diameter to total fiber diameter) were larger, indicating that myelin sheaths in Hom and Het were thinner than that in WT mice. (d) Quantitation analysis of axonal diameters showed that small sized axons were increased in L-MPZ Hom and Het mice compared to WT. **p*<0.05; ****p*<0.001 by one-way ANOVA with post-hoc Tukey's test. Data are presented as mean ± SD of experiments. *N* (mice) in WT = 6, Het= 6, Hom = 6.

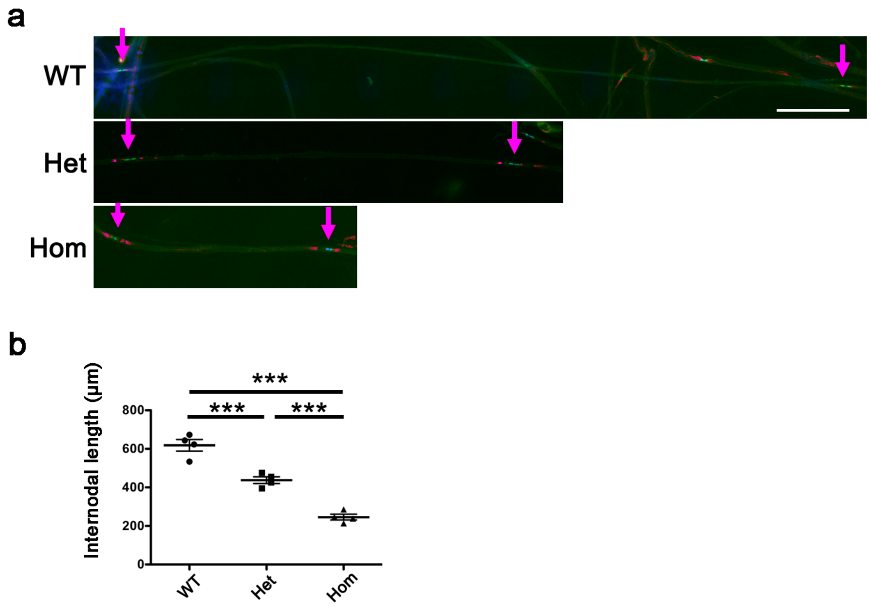

Supplementary Figure 5. Shorter myelin internodes in L-MPZ mouse sciatic nerves

(a) Triple-immunostaining of teased sciatic nerve fibers in 10-week-old L-MPZ WT, Het, Hom mice using anti-neurofascin (pan-NF, blue), anti-caspr (turquoise), and anti-Kv1.2 (magenta) antibodies. The node of Ranvier was indicated by magenta arrow. Full length of single internode was demonstrated between two neighboring nodes. Bar, 100 µm. (b) Measurement of the internodal length of each myelin (also see Supplementary Table 1). The internodal lengths were significantly decreased in L-MPZ Het and Hom mice. ****p*<0.001 by one-way ANOVA with post-hoc Tukey's test. Data are presented as mean ± SE of experiments. *N* (mice) in WT = 4, Het = 4, Hom = 4.

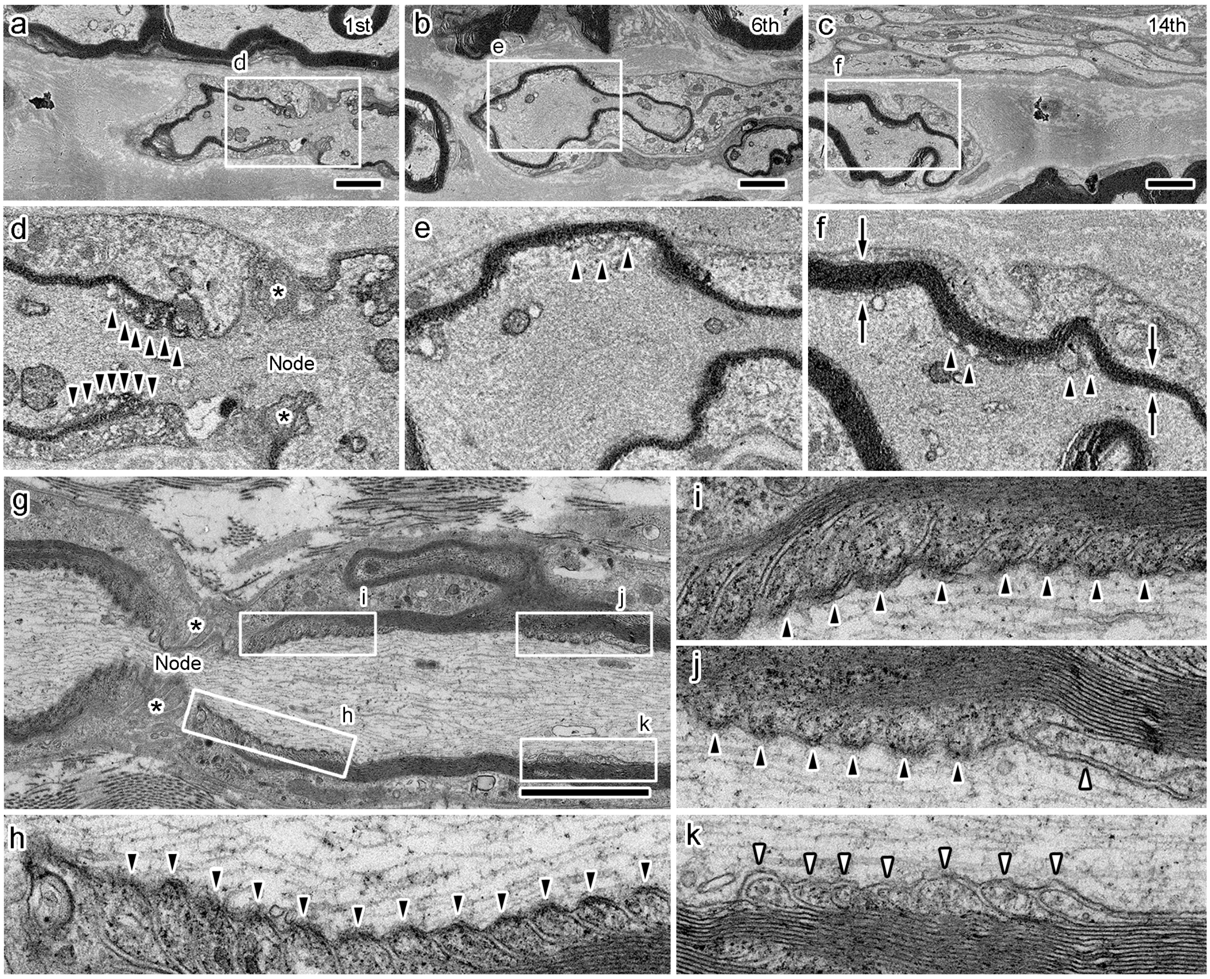

Supplementary Figure 6. Abnormal structures of paranodal regions in L-MPZ mice

(a–f) Representative serial images (1st, 6th, and 14th images are shown) of a single myelinated axon in the sciatic nerve of a 8-week-old L-MPZ Hom mouse. Groups of paranodal loops (d–f, arrowheads) intermittently appeared near nodal regions (d), and compact myelin sheath of different thickness flanked the groups of ectopic paranodal loops (f, arrows). (g–k) Transmission electron micrograph of a myelinated axon in L-MPZ Hom mice. The paranodal loops flanking the node (h, i, black arrowheads) and some ectopic paranodal loops (j, black arrowheads) had electron-dense transverse bands on the side facing the axolemma. Other ectopic paranodal loops lacked the electron-dense bands (j, k, white arrowheads). Indicated areas (a–c, g) were magnified (d–f, h–k). Asterisks (d, g) indicated nodal microvilli. Bar, 2 μm (a–c, g).

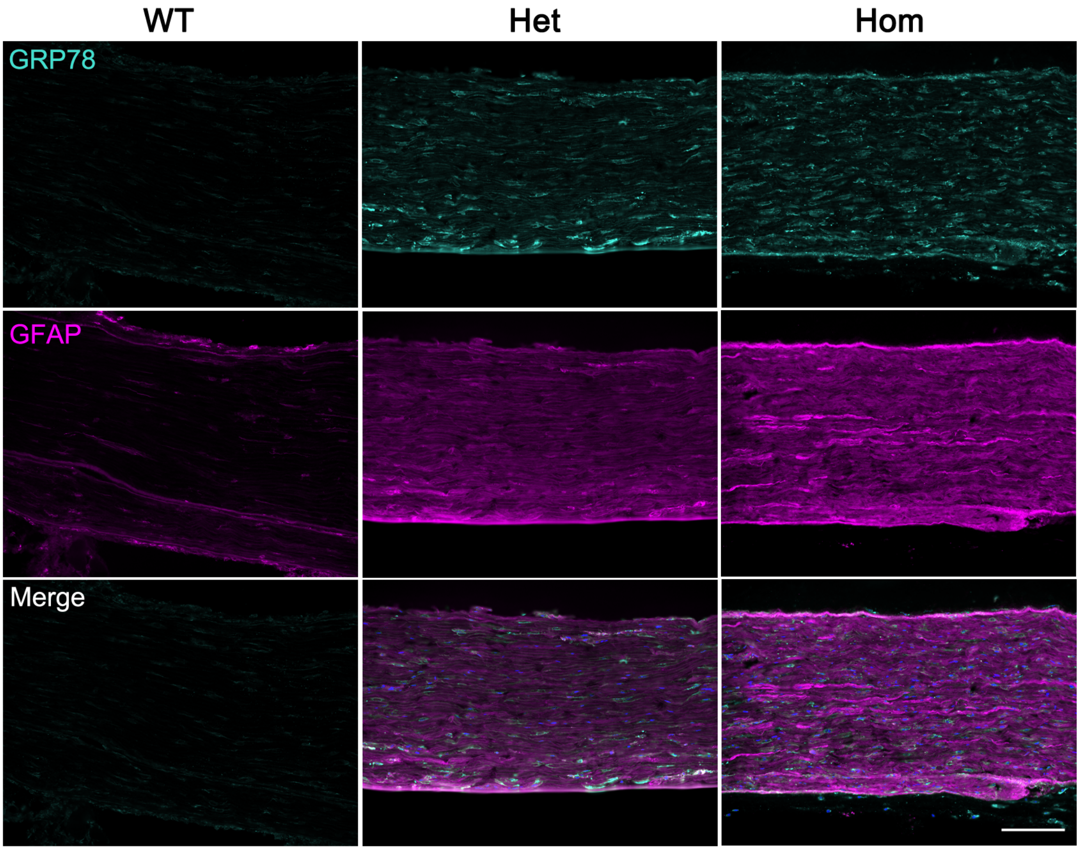

Supplementary Figure 7. Immunostaining of ER stress and non-myelinated Schwann cell markers

Representative immunostaining images of sciatic nerve sections of 10-week-old L-MPZ mice using ER stress marker anti-GRP78 (turquoise) and non-myelinated Schwann cell marker anti-GFAP (magenta) antibodies with DAPI (blue). GRP78- and GFAP-positive signals were apparently increased in L-MPZ Het and Hom mice. Bar, 100 μm.

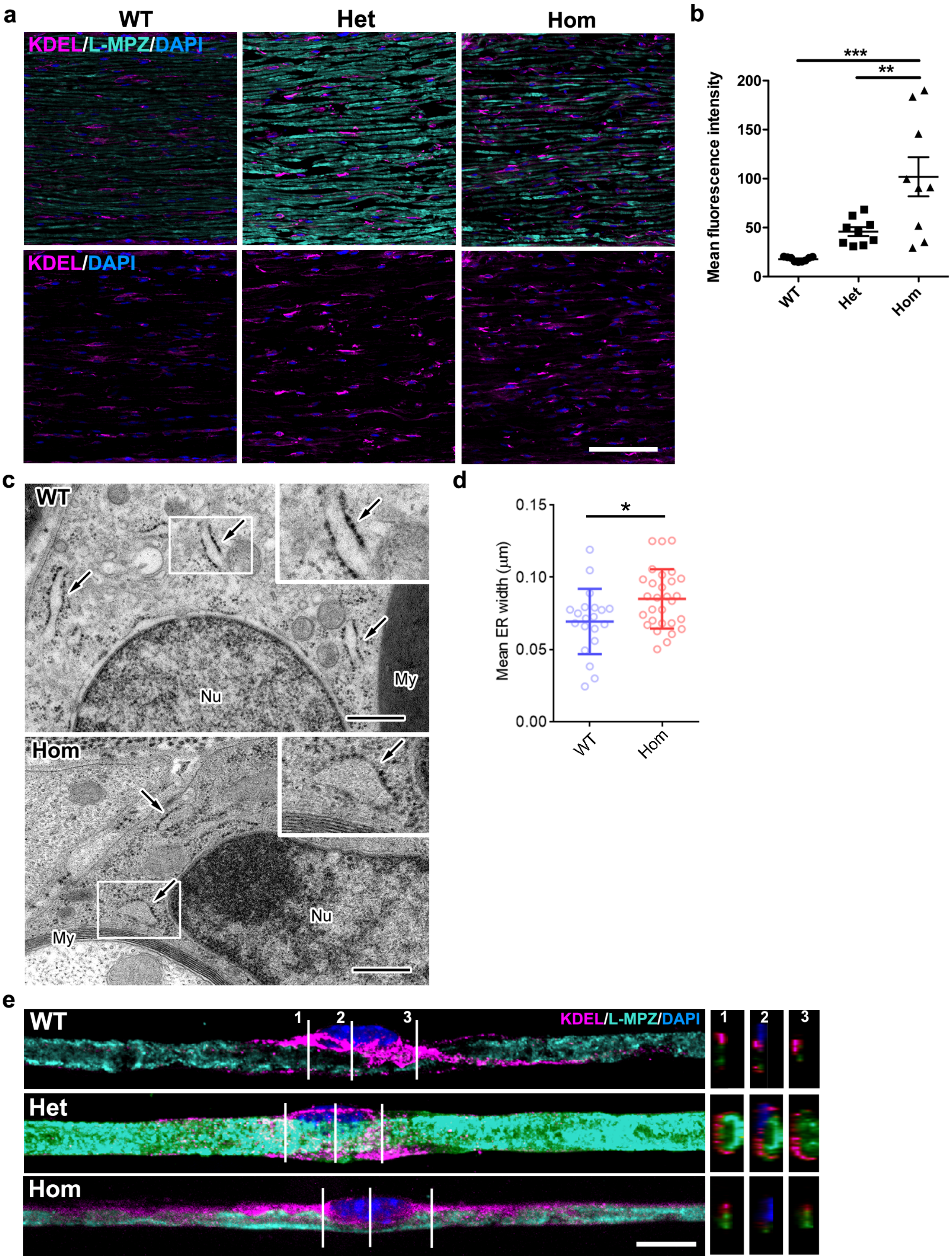
a
WT
Het
Hom
KDEL/L-MPZ/DAPI
KDEL/DAPI
b
***
**
Mean fluorescence intensity
200
150
100
50
0
WT
Het
Hom
c
WT
Nu
My
Hom
Nu
My
d
*
Mean ER width (μm)
0.15
0.10
0.05
0.00
WT
Hom
e
WT
Het
Hom
KDEL/L-MPZ/DAPI
1
2
3

Supplementary Figure 8. Increase of ER stress and swelling of ER in L-MPZ mice

(a) Representative double immunostaining images of sciatic nerve sections of 10-week-old L-MPZ mice using another ER stress marker anti-KDEL (magenta) and anti-L-MPZ (turquoise) antibodies with DAPI (blue). The lower row shows single KDEL-positive signals with DAPI signals. Bar, 100 μm. (b) For quantification of increase of anti-KDEL antigens, fluorescence intensities (au per μm^2, arbitrary unit per nerve area) of KDEL signals were measured from 3 confocal (X 40) fields of view in sections from each WT, Het or Hom mice. KDEL signal was significantly increased in L-MPZ Hom mice. **p<0.01; ***p<0.001 by one-way ANOVA with post-hoc Tukey's test. Data are presented as mean ± SE of experiments. *N* (mice) in WT = 3, Hom = 3. (c) Electron micrographs of myelinating Schwann cells in 8-week-old WT and L-MPZ Hom mice. Swelling of ER was observed in Schwann cells of L-MPZ mice. Indicated square areas were magnified in insets. Arrows, ER with membrane-bound ribosomes; Nu, nuclei; My, compact myelin. Bars, 500nm. (d) The graph shows the dot plot with means and SDs. Each dot represents the mean width obtained from one cell. *p<0.05 in Student's t-test. N= 20 (WT) or 29 (Hom) cells. (e) Representative double immunostaining images of teased sciatic nerve fibers of 10-week-old L-MPZ mice using anti-KDEL (magenta) and anti-L-MPZ (turquoise) antibodies with DAPI (blue). Z-stack images obtained from three positions were indicated at the right. Bar, 10 μm.

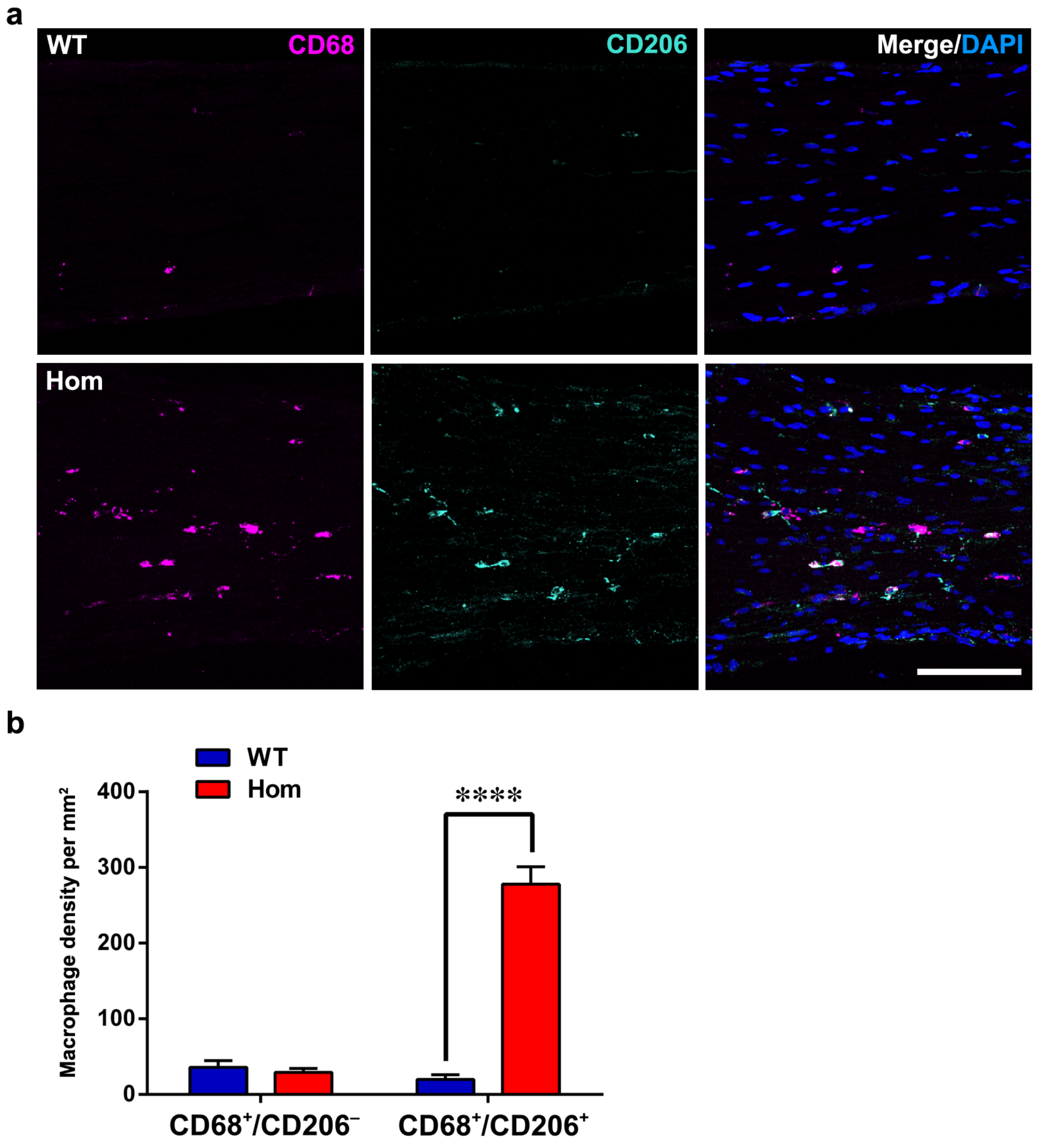

Supplementary Figure 9. Immunohistological typing of macrophages in L-MPZ mice

(a) Representative immunostaining images of sciatic nerve sections of 10-week-old L-MPZ mice using macrophage subtype marker anti-CD68 (magenta) and anti-CD206 (turquoise) antibodies with DAPI (blue). Bar, 100 μm. (b) For quantification, the number of $CD68^+/CD206^-$ or $CD68^+/CD206^+$ macrophages per mm^2 of each nerve was counted in 3 confocal (X40) fields of view in sections from each WT or Hom mice. The number of $CD68^+/CD206^+$ macrophage was significantly increased in L-MPZ Hom compared with WT mice. ****$p<0.0001$ by two-way ANOVA followed by Bonferroni multiple comparison tests. Data are presented as mean ± SE of experiments. *N* (mice) in WT = 3, Hom = 3.

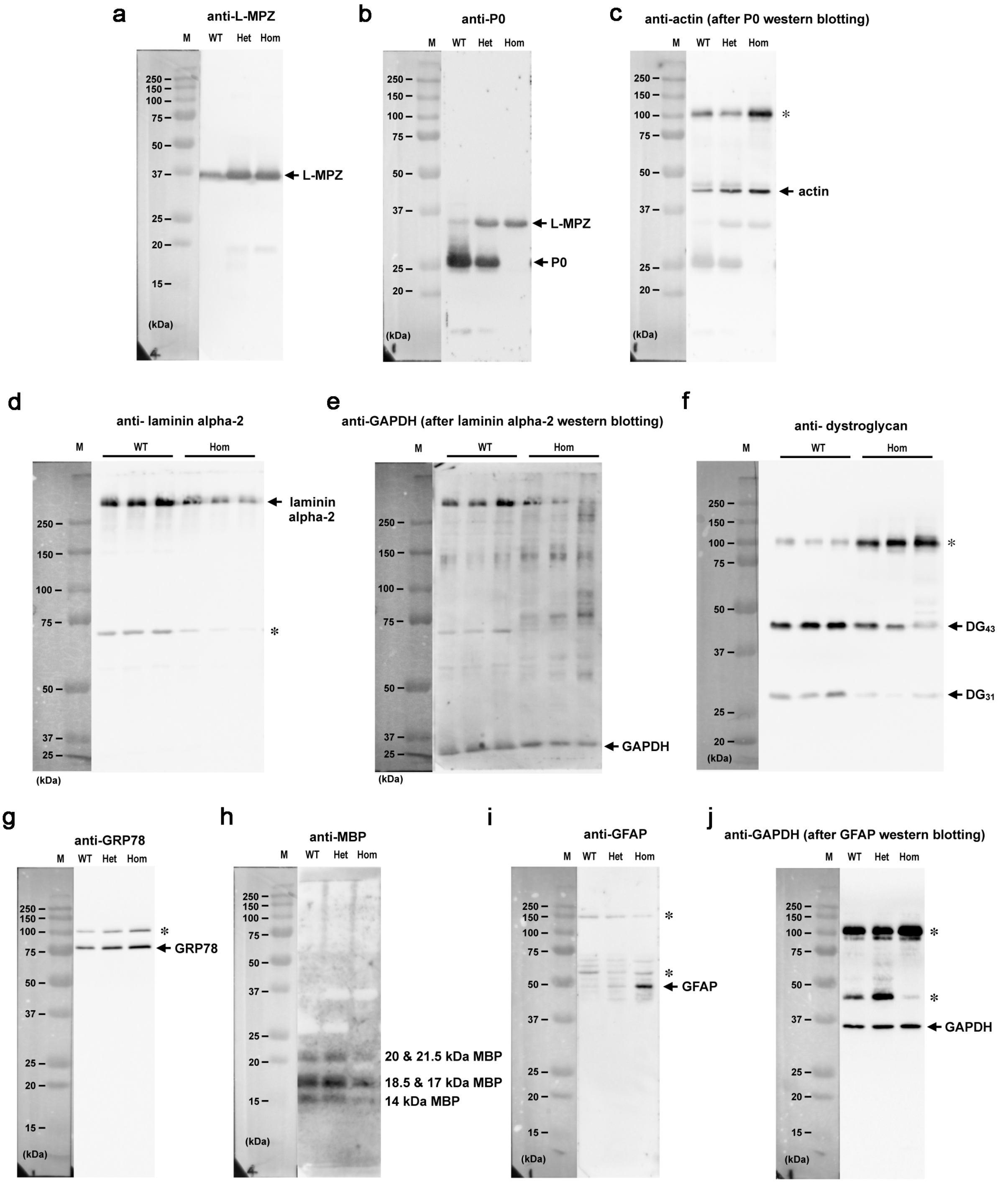

Supplementary Figure 10. Original western blot images for Figures
(a–c) Uncropped western blots for Supplementary Figure 1d. (d–f) Uncropped western blots for Supplementary Figure 3a. (g–j) Uncropped western blots for Figure 6a. Asterisks indicate nonspecific protein bands. M, molecular weight marker.

Supplementary Table 1. The phenotypic parameters of L-MPZ mice

Parameter	WT mice	L-MPZ Het mice	L-MPZ Hom mice
Tail suspension test early period (1-2 min) * (angle degree)	50.01 ± 3.78	42.32 ± 2.84	17.30 ± 4.00
Tail suspension test later period (4-5 min) * (angle degree)	39.52 ± 3.39	29.62 ± 2.24	7.840 ± 1.83
Rotarod (s) *	222.42 ± 23.47	84.44 ± 31.69	21.67 ± 5.32
NCV (m s^{-1}) *	34.87 ± 2.69	20.81 ± 1.26	11.78 ± 0.70
CMAP (mV) *	4.819 ± 0.35	3.062 ± 0.14	1.49 ± 0.093
f-ratio #	0.975 ± 0.079	1.381 ± 0.064	2.503 ± 0.16
Nodal length (μm) §	1.38 ± 0.03	1.82 ± 0.04	1.88 ± 0.04
Paranodal length (μm) §	4.103 ± 0.09	1.840 ± 0.17	1.76 ± 0.07
Juxtaparanodal length (μm) §	9.049 ± 0.33	24.81 ± 0.77	21.81 ± 0.93
Macrophages ($Iba1^+$) per mm^2 ¶	7.24 ± 1.40	96.85 ± 12.43	267.15 ± 12.69
Internodal length (μm) †	618.36 ± 30.08	437.80 ± 17.50	245.90 ± 14.87

Mean values with SEM of each phenotypical parameters in 10-week-old L-MPZ mice.
Experimental details are in Figure *1b–f, #2f, §5c–e, ¶7b, and †Supplementary Fig. 5b legends.

Supplementary Table 2. The EM analysis parameters of L-MPZ mice

Parameter	WT mice	L-MPZ Hom mice	P
G-ratio *	0.67 ± 0.01	0.73 ± 0.01	< 0.01
Axon diameter (μm) *	4.34 ± 0.13	3.13 ± 0.15	< 0.001
Axon without compact myelin (%) *	N	10.62 ± 1.93	< 0.001
Periodicity (nm) #	13.73 ± 0.15	14.75 ± 0.24	< 0.001
Macrophages per 10^6 μm^3 §	13.33 ± 5.85	66.66 ± 5.65	< 0.001

Mean values with SEM of each EM parameters in 8-week-old L-MPZ mice fibers.
Experimental details are in Figure *3e–h, #4f, and §7g legends. N, not observed.

Supplementary Table 3. The states around the node of Ranvier in L-MPZ mice nerves

state	WT	Het	Hom
Normal (%)	81.42 ± 1.45	24.49 ± 2.93	22.46 ± 3.19
Gap within paranodal staining (%)	6.48 ± 0.69	11.87 ± 3.69	18.10 ± 1.45
Gap between paranode and juxtaparanode (%)	1.74 ± 0.55	37.40 ± 3.25	39.64 ± 2.16
Absence of juxtaparanode (%)	10.36 ± 2.34	26.24 ± 3.86	19.80 ± 4.57

Mean values with SEM of each populations in 10-week-old L-MPZ mice fibers.

Experimental details are in Figure 5f legend.
