# Supplementary material for: Upregulation of large myelin protein zero leads to Charcot–Marie–Tooth disease-like neuropathy in mice
Source: Commun Biol. 2020 Mar 13;3:121. doi: 10.1038/s42003-020-0854-z (PMC7070019; doi:10.1038/s42003-020-0854-z)
Supplement: Supplementary file 2 — Description of Additional Supplementary Items [file 42003_2020_854_MOESM2_ESM.pdf]

Description of additional supplementary items

Supplementary Data 1
The source data underlying plots of Figs. 1–8 and Supplementary Figs. 3–5, 8, 9.

Supplementary Movie 1
Recorded video of tail suspension test of WT mouse during 1–2 min after start.

Supplementary Movie 2
Recorded video of tail suspension test of WT mouse during 4–5 min after start.

Supplementary Movie 3
Recorded video of tail suspension test of Het mouse during 1–2 min after start.

File Name: Supplementary Movie 4
Recorded video of tail suspension test of Het mouse during 4–5 min after start.

File Name: Supplementary Movie 5
Recorded video of tail suspension test of Hom mouse during 1–2 min after start.

File Name: Supplementary Movie 6
Recorded video of tail suspension test of Hom mouse during 4–5 min after start.
