# Supplementary material for: Upregulation of large myelin protein zero leads to Charcot–Marie–Tooth disease-like neuropathy in mice
Source: Commun Biol. 2020 Mar 13;3:121. doi: 10.1038/s42003-020-0854-z (PMC7070019; doi:10.1038/s42003-020-0854-z)
Supplement: Supplementary file 10 — Reporting Summary [file 42003_2020_854_MOESM10_ESM.pdf]

nature research

Corresponding author(s): Yoshihide Yamaguchi

Last updated by author(s): Feb 7, 2020

Reporting Summary

Nature Research wishes to improve the reproducibility of the work that we publish. This form provides structure for consistency and transparency in reporting. For further information on Nature Research policies, see Authors & Referees and the Editorial Policy Checklist.

Statistics

For all statistical analyses, confirm that the following items are present in the figure legend, table legend, main text, or Methods section.

n/a	Confirmed	
☐	☒	The exact sample size (*n*) for each experimental group/condition, given as a discrete number and unit of measurement
☐	☒	A statement on whether measurements were taken from distinct samples or whether the same sample was measured repeatedly
☐	☒	The statistical test(s) used AND whether they are one- or two-sided<br>*Only common tests should be described solely by name; describe more complex techniques in the Methods section.*
☒	☐	A description of all covariates tested
☐	☒	A description of any assumptions or corrections, such as tests of normality and adjustment for multiple comparisons
☐	☒	A full description of the statistical parameters including central tendency (e.g. means) or other basic estimates (e.g. regression coefficient) AND variation (e.g. standard deviation) or associated estimates of uncertainty (e.g. confidence intervals)
☐	☒	For null hypothesis testing, the test statistic (e.g. *F*, *t*, *r*) with confidence intervals, effect sizes, degrees of freedom and *P* value noted<br>*Give P values as exact values whenever suitable.*
☒	☐	For Bayesian analysis, information on the choice of priors and Markov chain Monte Carlo settings
☒	☐	For hierarchical and complex designs, identification of the appropriate level for tests and full reporting of outcomes
☒	☐	Estimates of effect sizes (e.g. Cohen's *d*, Pearson's *r*), indicating how they were calculated

Our web collection on statistics for biologists contains articles on many of the points above.

Software and code

Policy information about availability of computer code

Data collection	The source and references of all software were listed in the methods section.
Data analysis	Statistical analyses were performed by GraphPad Prism 5.

For manuscripts utilizing custom algorithms or software that are central to the research but not yet described in published literature, software must be made available to editors/reviewers. We strongly encourage code deposition in a community repository (e.g. GitHub). See the Nature Research guidelines for submitting code & software for further information.

Data

Policy information about availability of data

All manuscripts must include a data availability statement. This statement should provide the following information, where applicable:

- Accession codes, unique identifiers, or web links for publicly available datasets
- A list of figures that have associated raw data
- A description of any restrictions on data availability

All data generated during and/or analyzed during this study are included in this published article and its supplementary information. The source data underlying plots are presented in Supplementary Data 1. The detailed data supporting the findings of this study are available from the corresponding author upon reasonable request.

Field-specific reporting

Please select the one below that is the best fit for your research. If you are not sure, read the appropriate sections before making your selection.

☒ Life sciences ☐ Behavioural & social sciences ☐ Ecological, evolutionary & environmental sciences

For a reference copy of the document with all sections, see nature.com/documents/nr-reporting-summary-flat.pdf

Life sciences study design

All studies must disclose on these points even when the disclosure is negative.

Sample size	No statistical methods were used for determining sample sizes. Our sample sizes are similar to those previously reported in the field.
Data exclusions	We did not exclude data.
Replication	The number of animals and independently replicated experiments is described in the figure legends.
Randomization	Our data was not analysed with randomization, as similar experiments.
Blinding	Investigators were blinded during data collection and analysis.

Reporting for specific materials, systems and methods

We require information from authors about some types of materials, experimental systems and methods used in many studies. Here, indicate whether each material, system or method listed is relevant to your study. If you are not sure if a list item applies to your research, read the appropriate section before selecting a response.

Materials & experimental systems

n/a	Involved in the study	
☐	☒	Antibodies
☒	☐	Eukaryotic cell lines
☒	☐	Palaeontology
☐	☒	Animals and other organisms
☒	☐	Human research participants
☒	☐	Clinical data

Methods

n/a	Involved in the study	
☒	☐	ChIP-seq
☒	☐	Flow cytometry
☒	☐	MRI-based neuroimaging

Antibodies

Antibodies used

Rabbit polyclonal antibodies against:
L-MPZ, immunostaining, 1:4000; Western blotting, 1:40000
Kv1.2, Chemicon/Merck Millipore #AB5924, descontinued, immunostaining, 1:400
Glial Fibrillary Acidic Protein GFAP, DAKO/Agilent #IS524, immunostaining, 1:10; Western blotting, 1:1000
phospho-(Ser) PKC substrate Cell Signaling #2261S, Western blotting, 1:1000
DRP2, Sigma–Aldrich #HPA002949 DRP2, immunostaining, 1:50
GRP78 BiP, abcam #ab21685, immunostaining, 1:200; Western blotting, 1:2000
Iba1, Fujifilm Wako Pure Chemical Co. #019-19741, immunostaining, 1:800
Mannose receptor (CD206), abcam #ab64693, immunostaining, 1:200

Chicken polyclonal antibody against:
Neurofascin, R&D Systems/Minneapolis #AF3235, immunostaining, 1:400

Mouse monoclonal antibodies against:
Caspr, clone K65/35, UC Davis/NIH NeuroMab Facility, immunostaining, 1:400
sodium channel (Pan), clone K58/35, Sigma–Aldrich #S8809, immunostaining, 1:400
beta dystroglycan DAG43/DAG31, clone 43DAG1/8D5, Leica Biosystems #B-DG-CE, Western blotting, 1:1000
neurofilament, clone2F11, Nichirei Biosciences #41412551, immunostaining, 1:10
moesin, clone M22 (CR22), Sanko Junyaku, descontinued, immunostaining, 1:100
KDEL, clone 10C, Enzo Life Sciences, Inc. #ADI-SPA-827-F, immunostaining, 1:100
actin, Fujifilm Wako Pure Chemical Co. #012-27823, Western blotting, 1:10000
GAPDH, Fujifilm Wako Pure Chemical Co. #016-25523, Western blotting, 1:10000
CD68, clone ED1, abcam #ab31630, immunostaining, 1:1000

Rat monoclonal antibodies against:

MBP a.a. 82-87, Chemicon/Merck Millipore #MAB386, immunostaining, 1:200; Western blotting, 1:4000
laminin alpha-2, clone 4H8-2, Santa Cruz Biotechnology #sc-59854, Western blotting, 1:1000
E-cadherin, clone ECCD-2, Takara Bio #M108, immunostaining, 1:100

Goat polyclonal antibody against:
MPZ (P0), Abnova #PAB7332, Western blotting, 1:2000

Secondary antibodies for immunostaining:
Alexa 488- and 594-conjugated species-specific antibodies, Molecular Probes/Thermo Fisher Scientific 1:2000
aminomethyl coumarin-conjugated anti-chicken IgY antibody, Jackson ImmunoResearch Laboratories, 1:500

Secondary antibodies for Western blotting:
horseradish peroxidase-conjugated anti-mouse, anti-rabbit, anti-rat, and anti-goat IgG antibodies, Jackson ImmunoResearch Laboratories, 1:10000

Validation

The validation information for anti-L-MPZ antibody is cited in the methods section. The purchased antibodies are validated as shown in manufacture's website.

Verification for the descontined antibodies:

anti-KV1.2 #AB5924
Kourrich, S. et al. Dynamic interaction between sigma-1 receptor and Kv1.2 shapes neuronal and behavioral responses to cocaine. Cell 152, 236–247 (2013).
Lang, B. et al. Intracellular and non-neuronal targets of voltage-gated potassium channel complex antibodies. J. Neurol. Neurosurg. Psychiatry 88, 353–361 (2017).

anti-moesin, clone M22 (also known as CR22),
Hayashi, K., Yonemura, S., Matsui, T. & Tsukita, S. Immunofluorescence detection of ezrin/radixin/moesin (ERM) proteins with their carboxyl-terminal threonine phosphorylated in cultured cells and tissues. J. Cell. Sci. 112 (Pt 8), 1149–1158 (1999).

Animals and other organisms

Policy information about studies involving animals; ARRIVE guidelines recommended for reporting animal research

Laboratory animals

L-MPZ mice were generated by replacement of the canonical stop codon (TAG) with an alanine codon (GCG) using the CRISPR–Cas9 genome editing system49. L-MPZ founder mice were provided from the Laboratory Animal Resource Center, University of Tsukuba. For backcrossing, C57BL/6J mice were purchased from Charles River Laboratories Japan (Yokohama, Japan). Two founder mouse lines that transmitted the replaced genes to their offspring were produced and confirmed by genomic sequencing. L-MPZ mice were maintained on a C57BL/6J background. Genotypes were determined by Allele-specific PCR of mouse tail genomic DNA. Heterozygous animals were used for colony maintenance. Littermates or age-matched WT mice were used as control animals. Heterozygous animals were used for colony maintenance. Littermates or age-matched WT mice were used as control animals.

Wild animals

N/A

Field-collected samples

N/A

Ethics oversight

Mouse lines were maintained in a designated specific-pathogen-free environment at the animal facility of Tokyo University of Pharmacy and Life Sciences under university guidelines for the care and use of animals. All animal protocols were approved by the Institutional Animal Use Committee at Tokyo University of Pharmacy and Life Sciences (approval number: P16-95, P17-68, P18-76, and P19-46).

Note that full information on the approval of the study protocol must also be provided in the manuscript.
